# Identification of Predictive Biomarkers of Response to HSP90 Inhibitors in Lung Adenocarcinoma

**DOI:** 10.3390/ijms22052538

**Published:** 2021-03-03

**Authors:** Ángela Marrugal, Irene Ferrer, David Gómez-Sánchez, Álvaro Quintanal-Villalonga, María Dolores Pastor, Laura Ojeda, Luis Paz-Ares, Sonia Molina-Pinelo

**Affiliations:** 1 H12O-CNIO Lung Cancer Clinical Research Unit, Instituto de Investigación Hospital 12 de Octubre & Centro Nacional de Investigaciones Oncológicas (CNIO), 28029 Madrid, Spain; ammarrugal.imas12@h12o.es (Á.M.); iferrer@ext.cnio.es (I.F.); dags.imas12@h12o.es (D.G.-S.); lauojeda@ucm.es (L.O.); 2 CIBERONC, Respiratory Tract Tumors Program, 28029 Madrid, Spain; 3 Program in Molecular Pharmacology, Memorial Sloan Kettering Cancer Center, New York, NY 10065, USA; quintaa1@mskcc.org; 4 Institute of Biomedicine of Seville (IBIS) (HUVR, CSIC, Universidad de Sevilla), 41013 Sevilla, Spain; mpastor-ibis@us.es; 5 Medical Oncology Department, Hospital Universitario Doce de Octubre, 28041 Madrid, Spain; 6 Medical School, Universidad Complutense, 28040 Madrid, Spain

**Keywords:** HSP90 inhibitors, protein biomarkers predictive of response, lung adenocarcinoma

## Abstract

Heat shock protein 90 (HSP90) plays an essential role in lung adenocarcinoma, acting as a key chaperone involved in the correct functioning of numerous highly relevant protein drivers of this disease. To this end, HSP90 inhibitors have emerged as promising therapeutic strategies, even though responses to them have been limited to date. Given the need to maximize treatment efficacy, the objective of this study was to use isobaric tags for relative and absolute quantitation (iTRAQ)-based proteomic techniques to identify proteins in human lung adenocarcinoma cell lines whose basal abundances were correlated with response to HSP90 inhibitors (geldanamycin and radicicol derivatives). From the protein profiles identified according to response, the relationship between lactate dehydrogenase B (LDHB) and DNA topoisomerase 1 (TOP1) with respect to sensitivity and resistance, respectively, to geldanamycin derivatives is noteworthy. Likewise, rhotekin (RTKN) and decaprenyl diphosphate synthase subunit 2 (PDSS2) were correlated with sensitivity and resistance to radicicol derivatives. We also identified a relationship between resistance to HSP90 inhibition and the p53 pathway by glucose deprivation. In contrast, arginine biosynthesis was correlated with sensitivity to HSP90 inhibitors. Further study of these outcomes could enable the development of strategies to improve the clinical efficacy of HSP90 inhibition in patients with lung adenocarcinoma.

## 1. Introduction

Heat shock protein 90 (HSP90) is one of the most abundant and important heat shock proteins (HSPs) [1], functioning as a chaperone involved in the maturation, stabilization, and activation of more than 300 so-called “client proteins” [2,3]. Remarkably, most of HSP90′s client proteins are oncoproteins with crucial roles in different hallmarks of cancer [4,5]. For this reason, HSP90 is also known as the “cancer chaperone”, making it an important antitumor target, particularly in tumors such as lung cancer where HSP90 client proteins act as oncodrivers [6,7,8].

Lung cancer is the leading cause of cancer death in both men and women worldwide [9]. Concerning lung adenocarcinoma, the main non-small-cell lung cancer (NSCLC) subtype, which also represents approximately 50% of the total lung cancer cases, the fact that it can be driven by different genetic alterations is remarkable. Interestingly, the resulting proteins of many oncodrivers, such as epidermal growth factor receptor (EGFR) [10], Erb-B2 receptor tyrosine kinase 2 (ERBB2) [11], MET proto-oncogene, receptor tyrosine kinase (MET) [12], B-Raf proto-oncogene, serine/threonine kinase (BRAF) [13], or the echinoderm microtubule-associated protein-like (EML4)–anaplastic lymphoma kinase (ALK) fusion protein [14], are clients of HSP90. Nevertheless, the instability of these drivers and, as a consequence, their degradation via the targeted inhibition of HSP90 lead to a loss of viability of tumor cells. Consequently, elevated HSP90 expression has been correlated with a poorer clinical prognosis, such as resistance to chemotherapy and radiotherapy [15,16]. HSP90 inhibitors have shown antitumoral activity potential in preclinical studies and clinical trials in the treatment of NSCLC [17]. In addition, HSP90 inhibitors have been proposed to act as chemosensitizers [18] and radiosensitizers [19] and prevent acquired resistance to targeted therapies [20,21].

Some of the most promising HSP90 inhibitors have been the geldanamycin derivatives tanespimycin (17-AAG) and retaspimycin hydrochloride (IPI-504), as well as the radicicol analogs ganetespib (STA-9090) and luminespib (AUY-922) [22]. These inhibitors all act by blocking HSP90′s ATP binding site, which leads to proteasomal client degradation [23,24]. They have been widely evaluated in clinical trials with lung cancer patients and have shown encouraging results [25,26,27,28,29,30], specifically in those studies where the patients were molecularly stratified, such as lung adenocarcinoma patients with mutated EGFR and translocated ALK [25,27,28,31]. However, HSP90 inhibition as a treatment for lung cancer is still limited due to moderate efficacy outcomes, emergence of resistance, and early recurrence [17,32]. Recent research has focused on studying proteomic alterations produced at the cellular level after HSP90 inhibition as well as the physiological consequences of this blockade [33,34]. In this sense, previous results from our research group identified putative biomarkers of the HSP90 inhibition response in lung adenocarcinoma; these included eukaryotic translation initiation factor 3 subunit I (EIF3I) and transketolase, among others. In addition, it was shown that altered proteomic profiles after HSP90 inhibition affect energy production as well as metabolic and apoptotic pathways [35]. In this context, the complex interactome of HSP90 and the elevated number of cellular processes in which this chaperone is involved have made it commonplace for proteomic tools to be used to study HSP90 and its inhibition [36]. However, predictive biomarkers to identify which patients will respond positively (or not) to treatment have not yet been described, this being an essential condition for the therapeutic efficacy of HSP90 inhibitors to be optimized [32]. For these reasons, the main objective of our study was to use iTRAQ-based proteomics techniques to comprehensively characterize the proteomic profile associated with sensitivity and resistance to HSP90 inhibitors in human lung adenocarcinoma cell lines. Our findings are potentially important for improving therapeutic strategies to effectively treat this disease.

## 2. Results

### 2.1. Determination of Proteomic Profiles Correlated with HSP90 Inhibitor Sensitivity or Resistance

**Figure 1:**
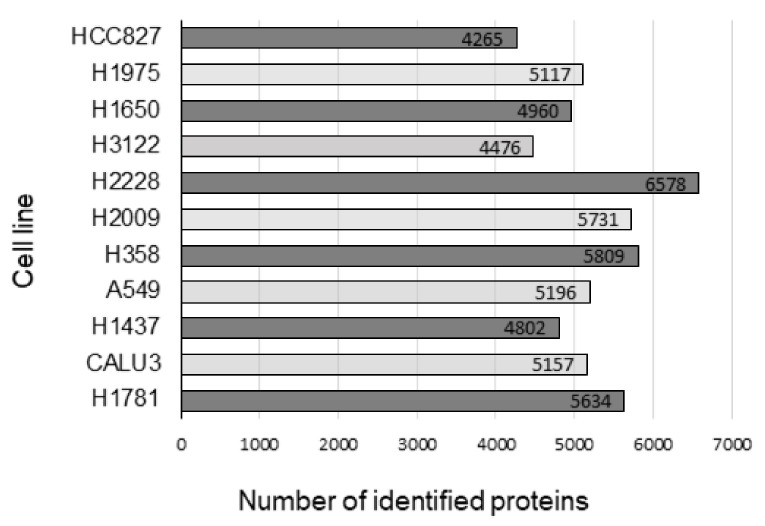
Number of proteins per cell line identified by iTRAQ and mass spectrometry analysis.

iTRAQ protein quantitation coupled to mass spectrometry (NanoLC-MS/MS, Thermo Fisher Scientific, Waltham, Massachusetts, United States) was employed to predict sensitivity or resistance to HSP90 inhibitors by means of proteomic profiling of a panel of lung adenocarcinoma cell lines. Prior to treatment, the cell lines were profiled as mentioned and the basal abundance of proteins was calculated. An average of around 5000 proteins per cell line was identified (Figure 1).

To evaluate the efficiency of the response to HSP90 inhibition, the IC_50_ values for four HSP90 inhibitors were determined in our cell line panel. Table 1 shows IC_50_ values for geldanamycin derivatives (17-AAG and IPI-504) and radicicol derivatives (STA-9090 and AUY-922). The IC_50_ value was used to select the three cell lines most sensitive and resistant to each HSP90 inhibitor studied. In response to 17-AAG, cell lines H1975, H1437, and H1650 showed the lowest IC_50_ values (ranging from 1.258 to 6.555 nM); in contrast, HCC827, H2009, and Calu-3 showed the highest values (IC_50_ values between 26.255 and 87.733 nM). For the other geldanamycin derivative, IPI-504, the most sensitive lines were H1437 (IC_50_: 3.473 nM), H1650 (IC_50_: 3.764 nM), and H358 (IC_50_: 4.662 nM), while the most resistant were H2009, Calu-3, and H2228 (IC_50_: 33.833, 43.295, and 46.340 nM, respectively). In response to the radicicol derivative STA-9090, the lowest IC_50_ values were found in cell lines H2228, H2009, and H1975 (range: 4.131–4.739 nM), and the highest values were found in H3122 (IC_50_: 7.991 nM), H1781 (IC_50_: 9.954 nM), and Calu-3 (IC_50_: 18.445). In response to AUY-922 treatment, H1650, H2009, and H1975 were the most sensitive cell lines (IC_50_ values ranging from 1.472 to 2.595 nM), while H1781, A549, and Calu-3 were the most resistant (IC_50_ values between 23.787 and 1740.91 nM).

Our next step was to identify proteins commonly appearing in cell lines that were more sensitive or more resistant to the different treatments. Spearman’s rank correlation coefficient was then used to identify whether the basal abundances of these common proteins were correlated with the IC_50_ value of each inhibitor used in all the studied cell lines. Based on this criterion, for the geldanamycin derivatives 17-AAG and IPI-504, 27 and 1664 proteins, respectively, were related to sensitivity, while 171 and 1772, respectively, were related to resistance. Conversely, for the radicicol analogs STA-9090 and AUY-922, 51 and 1294 proteins, respectively, were correlated with sensitivity, while 21 and 1257, respectively, were correlated with resistance (Table 2). Notably, more proteins were related to sensitivity to radicicol derivatives, while more proteins were related to resistance to geldanamycin derivatives. Based on these differences, the study was continued according to the family of HSP90 inhibitors used.

### 2.2. Identification of Candidate Biomarkers for Predicting Sensitivity to HSP90 Inhibitors in Lung Adenocarcinoma Cell Lines

Proteins whose basal abundances were correlated significantly with sensitivity to the different HSP90 inhibitor families were identified.

**Figure 2:**
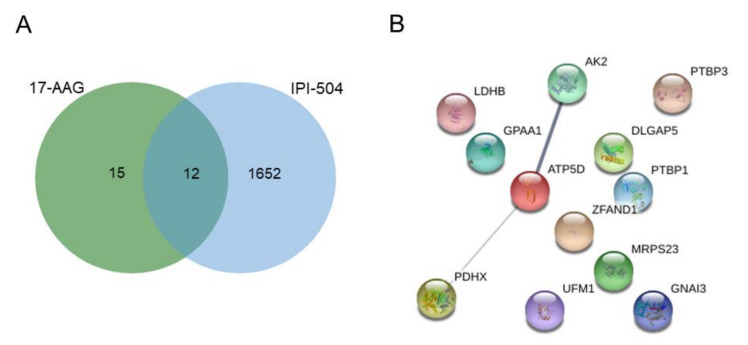
Study of proteins related to sensitivity to geldanamycin derivatives. (**A**) Venn diagram showing the overlap of proteins associated with a more sensitive response to 17-AAG and IPI-504 inhibitors. The analysis of shared proteins following geldanamycin treatments was performed with STRING software (https://string-db.org/), which identified (**B**) interactions between AK2, ATP5D, and PDHX proteins.

Focusing on the geldanamycin derivatives, a Venn diagram was built to determine the proteins associated with lower IC_50_ values for these HSP90 inhibitors (Figure 2A). We generated proteomic profiles for each drug, with 15 proteins expressed in only those cell lines with the highest sensitivity to 17-AAG and with 1652 proteins in the case of cell lines with high sensitivity to IPI-504 (Table S1). Additionally, both drugs shared 12 proteins in common that were associated with sensitivity to inhibition. Specifically, the 12 identified proteins were adenylate kinase 2 (AK2), ATP synthase subunit delta (ATP5D), disks large-associated protein 5 (DLGAP5), glycosylphosphatidylinositol anchor attachment 1 protein (GPAA1), guanine nucleotide-binding protein G(k) subunit alpha (GNAI3), lactate dehydrogenase B (LDHB), mitochondrial ribosomal protein S23 (MRPS23), polypyrimidine tract-binding protein 1 and 3 (PTBP1/3), pyruvate dehydrogenase protein X component (PDHX), ubiquitin-fold modifier 1 (UFM1), and zinc finger AN1-type containing 1 (ZFAND1) (Table S2). By mapping the protein–protein interactions of all common proteins associated with sensitivity to geldanamycin derivatives, we identified an interaction between AK2, ATP5D, and PDHX that is also associated with de novo purine synthesis (Figure 2B).

**Figure 3:**
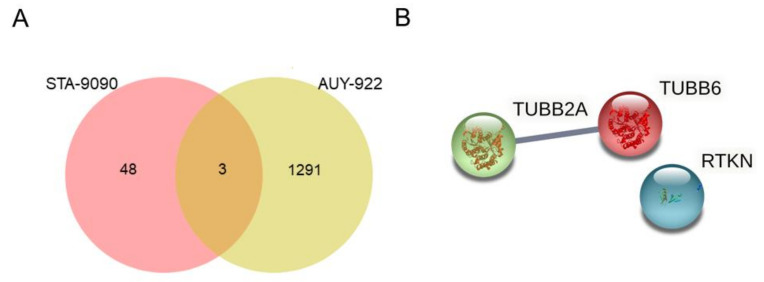
Analysis of proteins associated with sensitivity to radicicol derivatives. (**A**) Common proteins related to optimal response to STA-9090 and AUY-922 as well as those specific to each HPS90 inhibitor are shown in a Venn diagram. (**B**) STRING software was used to determine interactions between common proteins related to sensitivity to geldanamycin treatments.

Concerning radicicol analogs, rhotekin (RTKN), tubulin beta-2A chain (TUBB2A), and tubulin beta-6 chain (TUBB6) were identified as proteins related to sensitivity to both drugs tested (Figure 3A and Table S3). Figure 3B shows how, of all of the proteins identified, TUBB2A and TUBB6 were the only ones that interacted; this interaction is also associated with cytoskeletal regulation by the Rho GTPases pathway. In addition, we identified 48 proteins expressed only in cell lines with a high sensitivity to STA9090, while 1291 proteins were expressed only in cell lines sensitive to AUY-992 (Figure 3A and Table S1).

### 2.3. Detection of Potential Predictive Biomarkers of Resistance to HSP90 Inhibition in Lung Adenocarcinoma Cell Lines

**Figure 4:**
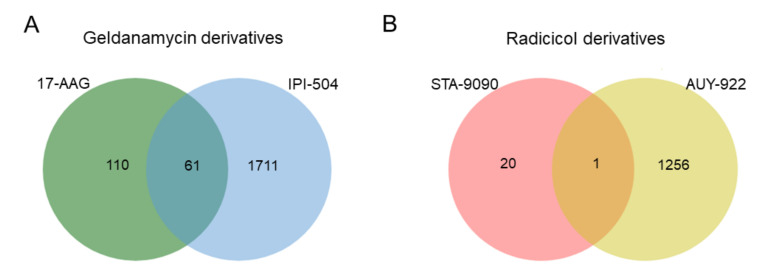
Venn diagrams showing the number of proteins related to resistance to specific geldanamycin (**A**) or radicicol (**B**) derivatives and overlap common to each HSP90 inhibitor family.

Proteins whose pretreatment expression was significantly correlated with a lack of response to each HSP90 inhibitor family were isolated and depicted in Venn diagram form (Figure 4).

The 61 proteins related to resistance to geldanamycin derivatives (Figure 4A) are presented in Table 3.

**Figure 5:**
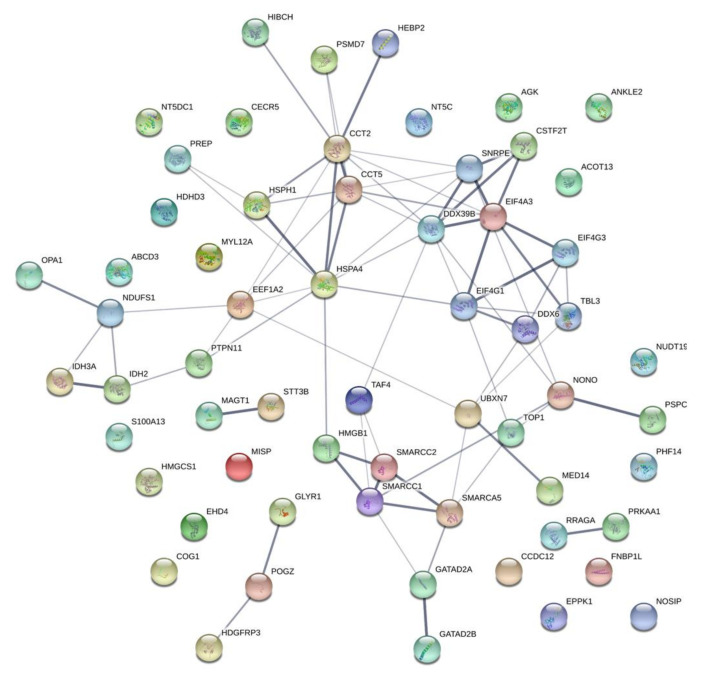
Map of interactions of common proteins related to geldanamycin resistance.

Among these proteins, DNA topoisomerase 1 (TOP1) which is involved in the DNA replication pathway, stands out. Figure 5 shows the large number of interactions between these 61 proteins. Interactions between several members of the SWI/SNF family (SWI/SNF complex subunit SMARCC1 (SMARCC1), SWI/SNF complex subunit SMARCC2 (SMARCC2), and SWI/SNF-related matrix-associated actin-dependent regulator of chromatin A5 (SMARCA5)) are noteworthy, along with resistance to these HSP90 inhibitors being related to the WNT signaling pathway. On the other hand, we also found 110 and 1711 proteins specifically expressed in cell lines exhibiting the most resistance to 17-AAG and IPI-504, respectively (Table S4).

With respect to proteins specifically expressed in response to STA-9090 and AUY-922 treatment, we identified 20 and 1256 proteins, respectively. Decaprenyl diphosphate synthase subunit 2 (PDSS2) was the only protein associated with resistance that was common to both inhibitors (Figure 4B and Table S4).

### 2.4. Candidate Pathways Associated with Susceptibility to HSP90 Inhibition

The PANTHER database was used to perform functional analyses of proteins significantly correlated with responses to HSP90 inhibitors. For a total of 58 biological pathways that were identified, only 6 (arginine biosynthesis, endogenous cannabinoid signaling, GABA-B receptor II signaling, gonadotropin-releasing hormone receptor pathway, opioid proenkephalin pathway, and opioid proopiomelanocortin pathway) were exclusively associated with sensitivity to the studied inhibitors. Interestingly, a greater number of pathways were related to resistance to HSP90 inhibition (Table 4). 

**Figure 6:**
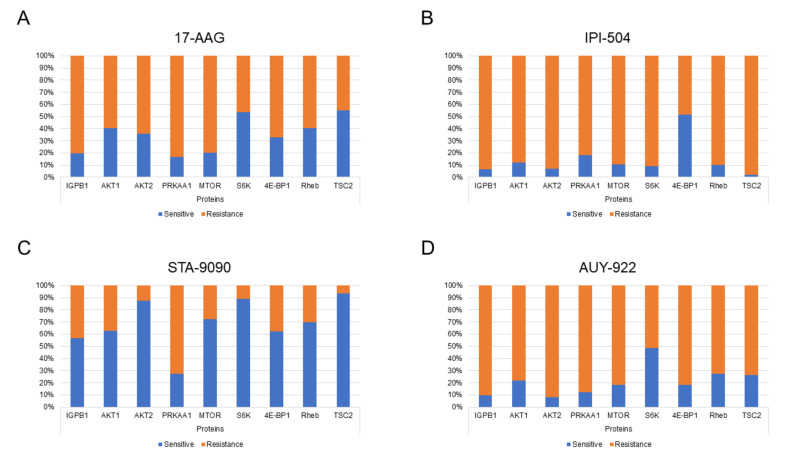
Basal abundance of proteins involved in the p53 pathway by glucose deprivation in cell lines sensitive or resistant to geldanamycin derivatives (17-AAG (**A**) and IPI-504 (**B**)) and radicicol derivatives (STA-9090 (**C**) and AUY-922 (**D**)).

Among them, the Toll receptor signaling pathway and the p53 pathway by glucose deprivation are relevant given their importance in tumor growth. Proteins involved in the p53 pathway by glucose deprivation identified in our work were immunoglobulin binding protein 1 (IGBP1), AKT serine/threonine kinase 1 (AKT1), AKT serine/threonine kinase 2 (AKT2), protein kinase AMP-activated catalytic subunit alpha 1 (PRKAA1), mechanistic target of rapamycin kinase (mTOR), ribosomal protein S6 kinase beta-1 (S6K), eukaryotic translation initiation factor 4E-binding protein 1 (4E-BP1), GTP-binding protein Rheb (Rheb), and tuberin (TSC2). Most proteins showed higher basal abundance in HSP90-inhibitor-resistant cell lines than in drug-sensitive ones, with the exception of STA-9090 (Figure 6).

## 3. Discussion

In this study, we used iTRAQ and mass spectrometry technology to identify proteins that served as predictive biomarkers describing sensitivity or resistance to HSP90 inhibition in lung adenocarcinoma cell lines. To this end, we analyzed the differential expression of specific proteins prior to and after inhibitor treatment and correlated our findings to the roles these proteins may play in signaling pathways involving tumorigenesis. Such an approach should enable us to optimize the manner in which HPS90 is used as a therapeutic target in lung cancer.

Treatment with the radicicol derivatives STA-9090 and AUY-922 revealed a large number of proteins significantly correlated with tumor cell sensitivity; this number was considerably greater than the number of proteins associated with resistance to these drugs. In contrast, more proteins related to resistance to the geldanamycin derivatives 17-AAG and IPI-504 were detected than those related to sensitivity. These data potentially indicate a greater efficacy of HSP90 inhibition through radicicol derivatives in lung adenocarcinoma cell lines and may explain why better clinical results have been achieved in lung cancer patients after treatment with radicicol derivatives [37], the only inhibitor family associated with ongoing clinical trials (NCT02503709 and NCT02535338).

Given differences in clinical results obtained according to the HSP90 inhibitor family used, this study focused on identifying biomarkers associated with the efficacy of geldanamycin and radicicol derivatives. As shown in our results, 12 proteins (AK2, ATP5D, DLGAP5, GPAA1, GNAI3, LDHB, MRPS23, PTBP1/3, PDHX, UFM1, and ZFAND1) were correlated with sensitivity to the geldanamycin analogs 17-AAG and IPI-504. Of these proteins, an association between LDHB and the antitumoral response has been proposed. Moreover, among the different isoenzymes of LDH, two of them—LDHB and LDHA—were reported as deregulated proteins in cancer cells and are involved in the regulation of tumor–stroma nutrient exchange [38]. A relationship between LDHB expression and response to other antitumor therapies, specifically adjuvant chemotherapy in patients with breast cancer, was also previously described [39]. Regarding NSCLC, it is noteworthy that the GALAXY-1 clinical trial showed that patients with high LDH expression responded better to a combination therapy consisting of the HSP90 inhibitor STA-9090 and docetaxel [29]. These data, despite not distinguishing between the A and B isoforms of LDH, together with the demonstrated importance of LDHB for the growth of certain lung adenocarcinomas [40], support the potential of LDHB as a predictive biomarker of the efficacy of geldanamycin derivatives. On the other hand, with respect to proteins related to resistance to the geldanamycin analogs 17-AAG and IPI-504, the presence of TOP1 among the 61 proteins identified was of note. In different tumor types, including lung cancer, a high expression of this important enzyme involved in DNA replication and transcription has been detected when compared to healthy tissue [41]. Furthermore, a low level of TOP1 expression has been proposed as a good prognostic factor related to greater overall survival in postoperative NSCLC patients [42]. These observations are consistent with results obtained in our work, where the TOP1 expression level and the DNA replication pathway in which it is involved are positively correlated with resistance to geldanamycin analogs. Based on this, and since different TOP1 inhibitors are used clinically for the treatment of small-cell lung, ovarian, and cervical cancer [43], their use in combination with 17-AAG or IPI-504 could be an interesting therapeutic strategy to be tested in lung adenocarcinoma patients presenting with high TOP1 expression. Our results also showed that Wnt signaling could be involved in the lack of response to 17-AAG and IPI-504 due to correlations between basal abundances of SMARCC1, SMARCC2, and SMARCA5 and resistance to geldanamycin derivatives. This pathway is involved in NSCLC initiation, progression, and metastasis [44,45]. Therefore, it is not surprising that a higher expression of SMARCC2 has been reported in tissue from NSCLC patients when compared to normal tissue [46]. Furthermore, in line with our data, SMARCC1 was previously described as a potential biomarker of efficacy with cytotoxic chemotherapy [47]. In addition, in bladder cancer, Li et al. observed decreased SMARCC1 expression levels after HSP90 inhibition, supporting our results [48]. Finally, it is worth highlighting that SMARCA5, which is overexpressed in different tumor types [49,50], forms a chromatin-remodeling complex with bromodomain adjacent to zinc finger domain 1B (BAZ1B) that facilitates the access of TOP1 at the replication fork. The overexpression of BAZ1B, and therefore that of SMARCA5, could potentiate the function of TOP1 during DNA replication, affecting the response of cancer cells to TOP1 inhibitors and to other treatments affecting replication [51]. These data might connect the two pathways proposed in this study, relating resistance to 17-AAG and IPI-504 with DNA replication and Wnt signaling. Taken together, further research is required on SMARCC1, SMARCC2, and SMARCA5, together with TOP1, as predictive biomarkers of resistance to geldanamycin derivatives as HSP90 inhibitors in lung adenocarcinoma.

Regarding radicicol derivatives, the relationship between the basal abundance of RTKN and its sensitivity to this family of HSP90 inhibitors is relevant. Among other functions, this Rho GTPase effector [52] is a key regulator of the cytoskeleton in multiple cellular functions, such as migration, adhesion, and cytokinesis [53]. Notably, our work identified cytoskeletal regulation by the Rho GTPases pathway as the only pathway associated with sensitivity to radicicol analogs in the studied lung adenocarcinoma cell lines. To this end, the radicicol derivative AUY-922 was shown to exert a strong antimigratory effect on tumor cells due primarily to reorganization of the cytoskeleton [54]. We, therefore, suggest that cytoskeletal properties and related HSP90 client proteins could be involved in favorable responses to the inhibition of this chaperone. On the other hand, among the proteins related to resistance to STA-9090 and AUY-922 treatments, a subunit of the enzyme decaprenyl diphosphate synthase, decaprenyl diphosphate synthase subunit 2 (PDSS2), plays a critical role in coenzyme Q10 (CoQ10) biosynthesis [55]. Low PDSS2 expression has been reported in different tumor types, including NSCLC, where the role of this protein as a tumor suppressor has been described [56,57]. However, research has demonstrated an association between CoQ10 and the decrease in the effectiveness of different chemotherapeutic agents in cancer cells due to the antioxidant activity of this coenzyme [58]. These observations support our results, where the basal abundance of PDSS2, and therefore CoQ10, was significantly correlated with resistance to radicicol derivatives in lung adenocarcinoma cell lines.

It is important to highlight that several proteins included in the identified protein pattern are involved in the HSP90 interactome [59]. There are unimpeachable pieces of evidence showing that the degradation of these client proteins is fundamental in the response to HSP90 inhibitors [8,24]; however, there is no information about their predictive role. Furthermore, the broad protein network of HSP90 includes not only its clients but also many proteins that, unlike clients, are overexpressed after its inhibition [48]. Nonetheless, due to the crucial role of the HSP90 interactome in the response to its inhibition, it is essential to know which proteins in our profile are clients of this chaperone [1]. Specifically, we found that ATP5D, TUBB2A, TUBB6, 26S proteasome non-ATPase regulatory subunit (7PSMD7), PRKAA1, conserved oligomeric Golgi complex subunit 1 (COG1), and myosin regulatory light chain 12A (MYL12A) are HSP90 client proteins with a potential predictive role of resistance or sensitivity to HSP90 inhibitors. Therefore, changes in these proteins, which are involved in the cytoskeleton, proteasome, ATP synthesis, or p53 pathway by glucose deprivation, among others, could be directly or indirectly affecting the response to the blockade of HSP90. Therefore, it would be essential to study these interesting proteins in depth to know how their basal abundances may be related, in addition to sensitivity or resistance to HSP90 inhibitors, with the response to them.

We also carried out a functional annotation of all proteins whose basal abundance was related to sensitivity or resistance to HSP90 inhibitors. Among all of the pathways correlated with sensitivity to HSP90 inhibition, the role of the arginine biosynthesis pathway is highly relevant, with this amino acid highly implicated in cell proliferation, as previously described [60]. Based on this premise, the potential role of argininosuccinate synthase 1 (ASS1) [61], a key enzyme in arginine biosynthesis and which we identified as a predictor of sensitivity to HSP90 inhibitors in lung adenocarcinoma, warrants attention. Moreover, among the pathways related to resistance to HSP90 inhibitors, we identified the relevance of p53 by glucose deprivation. Our analyses detected a higher basal abundance of nine proteins (IGPB1, AKT1, AKT2, PRKAA1, mTOR, S6K, 4E-BP1, Rheb, and TSC2) involved in the p53 pathway by glucose deprivation in resistant cell lines compared to those sensitive to HSP90 inhibitors. These results found for the majority of inhibitors studied, especially for AUY-922, were significant and in line with those of recent researchers who described this pathway as involved in the induction of cell death by altering multidrug resistance [62,63,64]. However, STA-9090 was the only inhibitor whose lack of response suggests that it must be related to the alteration of another pathway. Therefore, it is important to extend our knowledge and understanding of the proteins involved in this p53 by glucose deprivation pathway given their possible predictive role of response to HPS90 inhibition in lung adenocarcinoma.

Given that our results are based exclusively on in vitro data, the in vivo validation of potential biomarkers and proposed pathways related to the efficacy of HSP90 inhibition in lung adenocarcinoma will be the next important step to translate these findings into clinical practice. However, a limitation to this is the lack of patients undergoing treatment with HSP90 inhibitors, mainly due to the toxicity presented by clinically tested inhibitors and the lack of treatment response in patients with lung adenocarcinoma. The use of the proteins presented in this study as potential predictive biomarkers of response to inhibitors highlights important issues regarding the correct selection of lung adenocarcinoma patients, which would increase the potential benefits of HSP90 inhibition as a therapeutic strategy.

## 4. Materials and Methods

### 4.1. Cell Culture

Human lung adenocarcinoma cell lines (HCC827, H1975, H1650, H2009, H358, H2228, Calu-3, H1437, and H1781) were obtained from the American Type Culture Collection (ATCC). Dr. Sánchez and Dr. Koivunen kindly provided the A549 and H3122 cell lines, respectively. Cells were cultured in RPMI-1640 medium (Sigma-Aldrich, Saint Louis, MO, USA), with the exception of A549 and Calu-3 cells, which were propagated in DMEM (Sigma-Aldrich). All cell lines were supplemented with 10% fetal bovine serum (FBS, TICO Europe, Amstelveen, The Netherlands), 1% antibiotic–antimycotic solution (Sigma-Aldrich), and 1% glutamine. Cells were maintained at 37 °C in a humidified incubator under a 5% CO_2_ and 95% air atmosphere and subcultured every 2–3 days. Cells were authenticated and regularly checked for mycoplasma.

### 4.2. Cell Viability

Cells in the log phase were plated at 3 × 10^3^/well in 96-well plates for 24 h. HSP90 inhibitor derivatives of geldanamycin (tanespimycin (17-AAG) (Selleckchem, Munich, Germany) and retaspimycin hydrochloride (IPI-504) (Eurodiagnóstico, Madrid, Spain)) and radicicol derivatives (ganetespib (STA-9090) and luminespib (AUY-922) (Selleckchem, Munich, Germany)) were dissolved in dimethyl sulfoxide (DMSO) as stock solutions according to the manufacturers’ instructions. Cells were then independently treated with HSP90 inhibitors at concentrations ranging from 0.33 nM to 20 µM for 96 h. The half-maximal inhibitory concentration (IC_50_) for each inhibitor was determined from the dose–response curve. Three independent experiments were performed at each concentration.

### 4.3. Proteomic Sample Preparation

Each of the cell lines was grown in 10 cm diameter dishes for 24 h. Then, cells were collected, precipitated, and washed with ice-cold PBS. Next, the cells were resuspended in lysis buffer containing 1 M TEAB (triethylammonium bicarbonate, Sigma-Aldrich), 0.05% SDS, 1:100 phosphatase inhibitor cocktail (PhosSTOP EASYpack, Roche, Basel, Switzerland), 1:100 protease inhibitor cocktail (cOmplete Mini EDTA-free, Roche), and 0.002% benzonase (Novagen, Buenos Aires, Argentina) and lysed for 1 h on ice with intermittent vortexing. Cell debris was removed by centrifugation at 14,000 rpm for 15 min at 4 °C. Supernatants were collected, and protein concentrations assayed using Qubit fluorometric quantitation (Life Technologies, Carlsbad, CA, USA). Finally, 100 µg of each sample was aliquoted and stored at –80 °C until required. These lysates were prepared from each cell line and condition.

### 4.4. Trypsin Digestion and iTRAQ Labeling

The proteins from each sample were reduced with 50 mM TCEP (tris-(2-carboxyethyl)phosphine, AB Sciex) at 60 °C for 1 h with shaking and were subsequently alkylated with 200 mM MMTS (S-methyl methanethiosulfonate, AB Sciex) for 10 min at room temperature. Proteins were then trypsinized at 37 °C in a 10:1 ratio (*w/w*) of substrate/enzyme (Promega) in a water bath overnight. Finally, a speed-vac concentrator was used for drying the peptides.

The iTRAQ-labeling assay was conducted according to the manufacturer’s instructions (iTRAQ 8-plex, AB Sciex). Briefly, each tryptic digest was reconstituted in 1 M TEAB and labeled with one isobaric amine-reactive tag, with 113 being the focus in the present work. After 2 h of incubation, labeled samples were pooled, dried at 45 °C, and stored overnight at 4 °C.

### 4.5. Cleaning and Fractionation

iTRAQ-labeled samples were desalted using Oasis HLB C18 cartridges (Waters, Milford, MA, USA) and dried using a vacuum centrifuge. Peptides were then prefractionated using MCX Oasis columns (Waters) and increasing concentrations (50, 100, 200, 300, 400, 500, 600, 700, 800, and 900 mM and 1, 1.5, and 2 M) of ammonium formate. A total of 13 fractions were collected and individually washed using C18 cartridges, after which they were dried.

### 4.6. NanoLC-MS/MS Analysis

Peptides from each fraction were separated using nano-liquid chromatography (nano LC 1000, Thermo Scientific) and analyzed by means of nano-electrospray ionization (Proxeon Biosystems, Odense, Denmark) connected to a Q Exactive Plus Orbitrap mass spectrometer (Thermo Scientific). Briefly, 13 µL of each fraction was loaded, preconcentrated, and washed in an Acclaim PepMap (75 µm × 2 cm, nanoViper, C18, 3 µm, 100 Å) precolumn (Thermo Scientific). Peptides were separated in an analytical column (75 µm × 15 cm, nanoViper, C18, 2 µm, 100 Å (Acclaim PepMap RSLC)) (Thermo Scientific) for 240 min at 200 nL/min.

Peptides were eluted with a gradient of buffer A (0.1% formic acid, 100% H_2_O) to buffer B (0.1% formic acid, 100% acetonitrile) as follows: 0–220 min, 0–35% buffer B; 220–230 min, 35–45% buffer B; and 230–240 min, 45–95% buffer B. The Q Exactive system was used for MS/MS analysis in the positive ion and information-dependent acquisition modes. Mass spectra were acquired over a scan range of 200 to 1800 *m/z* with a resolution of 70,000 FWHM from *m/z* 100. Up to 15 precursors with a charge state ≥2 were selected and incorporated into an exclusion list for 60 s. Peptide identification and quantification were carried out considering the higher-energy collisional dissociation (HCD) spectrum. HCD fragmentation was performed with a collision energy of 32% to maximize the abundance of iTRAQ reporter ions.

### 4.7. Protein Identification and Quantification

Peptide identification and quantification were conducted using the Sequest HT search engine and Percolator, both of which were included in Proteome Discoverer 1.4 software (Thermo Fisher Scientific). Each MS/MS spectrum was searched against the UniProt Database for *Homo sapiens*. The parameter considered for the search was digestion enzyme (trypsin); iTRAQ 8-plex peptide label (*N*-terminal) and iTRAQ 8-plex peptide label (lysine) were used as fixed modifications, and the oxidation of methionine and carbamidomethylation of cysteine were selected as variable modifications. Subsequently, the relative peptide abundances were determined using the MS/MS scans of iTRAQ-labeled peptides, where the ratios of peak areas of the iTRAQ reporter ions reflect the relative abundances of the peptides and therefore of proteins in the samples. Proteins had to contain at least two unique peptides with a significance score ≥95%, a ratio with a *p*-value ≤ 0.05, and a false discovery rate (FDR) <2 to be considered quantifiable.

### 4.8. Data Analysis

To identify potential predictive proteins related to sensitivity or resistance to HSP90 inhibitors, we analyzed the proteomic signature in human lung adenocarcinoma cells prior to treatment and correlated this with the efficacy of the response to treatment. The sum of the intensities of the peptides identified in the control group labeled with 113 was employed to calculate the basal abundances of proteins in the pretreated cell lines. On the other hand, those proteins commonly identified in the three cell lines most sensitive or resistant to each HSP90 inhibitor were selected. Then, for each of these proteins of interest, the Spearman coefficient was employed to test for a potential correlation between the basal abundance and the IC_50_ value of each inhibitor in all of the cell lines where these proteins were identified. Only proteins with a significant correlation (*p*-value < 0.05) were used in the subsequent analysis.

Sensitivity- or resistance-related proteins found in common for each HSP90 inhibitor family were grouped in Venn–Euler diagrams using the jvenn (http://jvenn.toulouse.inra.fr/app/index.html) program. Next, proteins of interest were functionally analyzed and categorized according to their biological processes and molecular functions using the PANTHER (Protein ANalysis THrough Evolutionary Relationships) online database (http://pantherdb.org/ accessed on May 2020). Finally, known and predicted protein–protein interaction networks of proteins were built based on the publicly available Search Tool for the Retrieval of Interacting Genes/Proteins (STRING) database (https://string-db.org/ accessed on May 2020). Only results with a Benjamini–Hochberg adjusted *p*-value of less than 0.05 were considered statistically significant.

## 5. Conclusions

We analyzed proteins whose basal abundances were correlated with response to HSP90 inhibitors in a panel of lung adenocarcinoma cell lines using iTRAQ-based assays. A total of 3219 proteins were associated with sensitivity to treatment with HSP90 inhibitors, and 3384 proteins were associated with resistance to treatment with HSP90 inhibitors. It is relevant to highlight that among all the proteins identified in our predictive profile, seven of them are HSP90 client proteins, which could be indicating an ensured effect of inhibition. In addition, we found specific differences according to the two families of inhibitors used; this was highlighted by the roles of LDHB and TOP1 proteins in sensitivity and resistance to geldanamycin analogs, respectively. In the case of radicicol derivatives, RTKN was correlated with sensitivity to these treatments, and PDSS2 was correlated with resistance to these treatments. In addition, functional annotation analyses of identified proteins revealed that the p53 pathway by glucose deprivation and arginine biosynthesis were correlated with resistance and sensitivity to HSP90 inhibitors, respectively. A thorough in vivo validation of our findings is required for future clinical use of these inhibitors to treat human lung adenocarcinoma.

## Figures and Tables

**Table 1:** IC_50_ values for geldanamycin and radicicol analogs.

Cell Line	Geldanamycin Derivatives		Radicicol Derivatives	
	IC_50_ 17-AAG (nM)	IC_50_ IPI-504 (nM)	IC_50_ STA-9090 (nM)	IC_50_ AUY-922 (nM)
HCC827	26.255	17.145	5.138	4.167
H1975	1.258	12.750	4.739	2.595
H1650	6.554	3.764	5.659	1.472
H3122	26.165	28.371	7.991	9.110
H2228	10.888	46.340	4.131	4.488
H2009	43.198	33.833	4.659	2.477
H358	13.066	4.662	7.740	8.105
A549	16.296	19.492	6.310	30.733
H1437	3.708	3.473	6.794	2.814
CALU-3	87.733	43.295	18.445	1740.91
H1781	12.345	30.975	9.954	23.787

**Table 2:** Number of proteins significantly correlated with sensitivity or resistance to the studied HSP90 inhibitors.

Treatment	Response	Number of Response-Related Proteins	
		Detected	Significant
17-AAG	Sensitivity	3724	27
	Resistance	3607	171
IPI-504	Sensitivity	3898	1664
	Resistance	4395	1772
STA-9090	Sensitivity	4497	51
	Resistance	3704	21
AUY-922	Sensitivity	4149	1294
	Resistance	3934	1257

**Table 3:** Proteins related to a shared resistance to 17-AAG and IPI-504.

Protein Name	UniProt ^1^	Gene ^2^
Spliceosome RNA helicase DDX39B	Q13838	*DDX39B*
26S proteasome non-ATPase regulatory subunit 7	P51665	*PSMD7*
3-hydroxyisobutyryl-CoA hydrolase, mitochondrial	Q6NVY1	*HIBCH*
5′(3′)-deoxyribonucleotidase, cytosolic type	Q8TCD5	*NT5C*
5′-AMP-activated protein kinase catalytic subunit alpha-1	Q13131	*PRKAA1*
5′-nucleotidase domain containing 1	Q5TFE4	*NT5DC1*
Acyl-coenzyme A thioesterase 13	Q9NPJ3	*ACOT13*
Acylglycerol kinase, mitochondrial	Q53H12	*AGK*
Ankyrin repeat and LEM domain-containing protein 2	Q86XL3	*ANKLE2*
ATP-binding cassette sub-family D member 3	P28288	*ABCD3*
Cleavage stimulation factor subunit 2 tau variant	Q9H0L4	*CSTF2T*
Coiled-coil domain containing 12	Q8WUD4	*CCDC12*
Conserved oligomeric Golgi complex subunit 1	Q8WTW3	*COG1*
DNA topoisomerase 1	P11387	*TOP1*
Dolichyl-diphosphooligosaccharide--protein glycosyltransferase subunit STT3B	Q8TCJ2	*STT3B*
Dynamin-like 120 kDa protein, mitochondrial	O60313	*OPA1*
EH domain-containing protein 4	Q9H223	*EHD4*
Elongation factor 1-alpha 2	Q05639	*EEF1A2*
Epiplakin	P58107	*EPPK1*
Eukaryotic initiation factor 4A-III	P38919	*EIF4A3*
Eukaryotic translation initiation factor 4 gamma 1	Q04637	*EIF4G1*
Eukaryotic translation initiation factor 4 gamma 3	O43432	*EIF4G3*
Formin-binding protein 1-like	Q5T0N5	*FNBP1L*
Haloacid dehalogenase like hydrolase domain containing 3	Q9BSH5	*HDHD3*
Haloacid dehalogenase-like hydrolase domain-containing 5	Q9BXW7	*CECR5*
Heat shock protein 105 kDa and 70 kDa proteins	Q92598	*HSPH1*
Heat shock protein family A member 4	P34932	*HSPA4*
Heme-binding protein 2	Q9Y5Z4	*HEBP2*
Hepatoma-derived growth factor-related protein 3	Q9Y3E1	*HDGFRP3*
High mobility group protein B1	P09429	*HMGB1*
Hydroxymethylglutaryl-CoA synthase, cytoplasmic	Q01581	*HMGCS1*
Isocitrate dehydrogenase [NAD] subunit alpha, mitochondrial	P50213	*IDH3A*
Isocitrate dehydrogenase [NADP], mitochondrial	P48735	*IDH2*
Magnesium transporter protein 1	Q9H0U3	*MAGT1*
Mediator of RNA polymerase II transcription subunit 14	O60244	*MED14*
Mitotic interactor and substrate of PLK1	Q8IVT2	*MISP*
Myosin regulatory light chain 12A	P19105	*MYL12A*
NADH-ubiquinone oxidoreductase 75 kDa subunit, mitochondrial	P28331	*NDUFS1*
Nitric oxide synthase-interacting protein	Q9Y314	*NOSIP*
Non-POU domain-containing octamer-binding protein	Q15233	*NONO*
Nucleoside diphosphate-linked moiety X motif 19	A8MXV4	*NUDT19*
Paraspeckle component 1	Q8WXF1	*PSPC1*
PHD finger protein 14	O94880	*PHF14*
Pogo transposable element with ZNF domain	Q7Z3K3	*POGZ*
Probable ATP-dependent RNA helicase DDX6	P26196	*DDX6*
Prolyl endopeptidase	P48147	*PREP*
Protein S100-A13	Q99584	*S100A13*
Putative oxidoreductase GLYR1	Q49A26	*GLYR1*
Ras-related GTP-binding protein A	Q7L523	*RRAGA*
Small nuclear ribonucleoprotein E	P62304	*SNRPE*
SWI/SNF complex subunit SMARCC1	Q92922	*SMARCC1*
SWI/SNF complex subunit SMARCC2	Q8TAQ2	*SMARCC2*
SWI/SNF-related matrix-associated actin-dependent regulator of chromatin subfamily A member 5	O60264	*SMARCA5*
T-complex protein 1 subunit beta;	P78371	*CCT2*
T-complex protein 1 subunit epsilon	P48643	*CCT5*
Transcription initiation factor TFIID subunit 4	O00268	*TAF4*
Transcriptional repressor p66-alpha	Q86YP4	*GATAD2A*
Transcriptional repressor p66-beta	Q8WXI9	*GATAD2B*
Transducin beta-like protein 3;	Q12788	*TBL3*
Tyrosine-protein phosphatase non-receptor type 11	Q06124	*PTPN11*
UBX domain-containing protein 7	O94888	*UBXN7*

^1^ UniProt accession number. ^2^ Entrez gene ID.

**Table 4:** Pathways related to responses to HSP90 inhibitors.

Sensitivity	Resistance
Arginine biosynthesis	Adrenaline and noradrenaline biosynthesis
Endogenous cannabinoid signaling	Alzheimer’s disease–amyloid secretase pathway
GABA-B receptor II signaling	Blood coagulation
Gonadotropin-releasing hormone receptor pathway	Coenzyme A biosynthesis
Opioid proenkephalin pathway	General transcription regulation
Opioid proopiomelanocortin pathway	Ornithine degradation
	p53 pathway by glucose deprivation
	Purine metabolism
	Toll receptor signaling pathway
	Vasopressin synthesis

## Data Availability

Not applicable.

## Supplementary Materials

Supplementary materials can be found at https://www.mdpi.com/1422-0067/22/5/2538/s1. Table S1. Proteins identified in human cell lines from lung adenocarcinoma with the highest sensitivity to each geldanamycin derivative, Table S2. Protein profile associated with sensitivity to both geldanamycin derivatives in lung adenocarcinoma, Table S3. Proteins related to sensitivity to radicicol analogs in lung adenocarcinoma, Table S4. Proteins specifically expressed in lung adenocarcinoma cell lines with the highest resistance to 17-AAG and IPI-504.

## Abbreviations

17-AAG	Tanespimycin
7PSMD7	26S proteasome non-ATPase regulatory subunit
4E-BP1	Eukaryotic translation initiation factor 4E-binding protein 1
AK2	Adenylate kinase 2
AKT1	AKT serine/threonine kinase 1
AKT2	AKT serine/threonine kinase 2
ALK	Anaplastic lymphoma kinase
ATP5D	ATP synthase subunit delta
AUY-922	Luminespib
BRAF	B-Raf proto-oncogene: serine/threonine kinase
COG1	Conserved oligomeric Golgi complex subunit 1
DLGAP5	Disks large-associated protein 5
EGFR	Epidermal growth factor receptor
EIF3I	Eukaryotic translation initiation factor 3 subunit I
EML4	Echinoderm microtubule-associated protein-like
ERBB2	Erb-B2 receptor tyrosine kinase 2
GNAI3	Guanine nucleotide-binding protein G(k) subunit alpha
GPAA1	Glycosylphosphatidylinositol anchor attachment 1 protein
HSP	Heat shock protein
HSP90	Heat shock protein 90
IGBP1	Immunoglobulin binding protein 1
IPI-504	Retaspimycin hydrochloride
LDHB	Lactate dehydrogenase B
MET	MET proto-oncogene, receptor tyrosine kinase
MYL12A	Myosin regulatory light chain 12A
MRPS23	Mitochondrial ribosomal protein S23
mTOR	Mechanistic target of rapamycin kinase
NSCLC	Non-small-cell lung cancer
PDHX	Pyruvate dehydrogenase protein X component
PDSS2	Decaprenyl diphosphate synthase subunit 2
PRKAA1	Protein kinase AMP-activated catalytic subunit alpha 1
PTBP1	Polypyrimidine tract-binding protein 1
PTBP3	Polypyrimidine tract-binding protein 3
Rheb	GTP-binding protein Rheb
RTKN	Rhotekin
S6K	Ribosomal protein S6 kinase beta-1
STA-9090	Ganetespib
TOP1	DNA topoisomerase 1
TSC2	Tuberin
TUBB2A	Tubulin beta-2A chain
TUBB6	Tubulin beta-6 chain
UFM1	Ubiquitin-fold modifier 1
ZFAND1	Zinc finger AN1-type containing 1
