# Supplementary material for: Identification of Predictive Biomarkers of Response to HSP90 Inhibitors in Lung Adenocarcinoma
Source: Int J Mol Sci. 2021 Mar 3;22(5):2538. doi: 10.3390/ijms22052538 (PMC7962034; doi:10.3390/ijms22052538)
Supplement: Supplementary file 1 [file ijms-22-02538-s001.zip › B.SupplementaryTable_2_Common proteins related to senstivity to GA inhibitors.docx]

| **Protein Name** | **UniProt^1^** | **Gene^2^** |
| --- | --- | --- |
| Adenylate kinase 2, mitochondrial | P54819 | AK2 |
| ATP synthase subunit delta, mitochondrial | P30049 | ATP5D |
| Disks large-associated protein 5 | Q15398 | DLGAP5 |
| Glycosylphosphatidylinositol anchor attachment 1 protein | O43292 | GPAA1 |
| Guanine nucleotide-binding protein G(k) subunit alpha | P08754 | GNAI3 |
| Lactate dehydrogenase B | P07195 | LDHB |
| Mitochondrial ribosomal protein S23 | Q9Y3D9 | MRPS23 |
| Polypyrimidine tract-binding protein 1 | P26599 | PTBP1 |
| Polypyrimidine tract-binding protein 3 | O95758 | PTBP3 |
| Pyruvate dehydrogenase protein X component, mitochondrial | O00330 | PDHX |
| Ubiquitin-fold modifier 1 | P61960 | UFM1 |
| Zinc finger AN1-type containing 1 | Q8TCF1 | ZFAND1 |

1= UniProt Accession Number; 2= Gene Symbol
