# Supplementary material for: Identification of Predictive Biomarkers of Response to HSP90 Inhibitors in Lung Adenocarcinoma
Source: Int J Mol Sci. 2021 Mar 3;22(5):2538. doi: 10.3390/ijms22052538 (PMC7962034; doi:10.3390/ijms22052538)
Supplement: Supplementary file 1 [file ijms-22-02538-s001.zip › C.SupplementaryTable3_Common proteins related to sentitivity to RD inhibitors.docx]

| **Protein Name** | **UniProt^1^** | **Gene^2^** |
| --- | --- | --- |
| Rhotekin | Q9BST9 | RTKN |
| Tubulin beta-2A chain | Q13885 | TUBB2A |
| Tubulin beta-6 chain | Q9BUF5 | TUBB6 |

1= UniProt Accession Number; 2= Gene Symbol
